# Tension at the Surface: Which Phase Is More Important, Liquid or Vapor?

**DOI:** 10.1371/journal.pone.0008281

**Published:** 2009-12-14

**Authors:** Andrew M. Prpich, Yuebiao Sheng, Wei Wang, M. Elias Biswas, P. Chen

**Affiliations:** 1 Department of Chemical Engineering, University of Waterloo, Waterloo, Ontario, Canada; 2 National Laboratory of Solid State Microstructure, Institute of Biophysics, and Department of Physics, Nanjing University, Nanjing, China; 3 Department of Chemical and Biomolecular Engineering, Johns Hopkins University, Baltimore, Maryland, United States of America; The Research Institute for Children, United States of America

## Abstract

Tension at the surface is a most fundamental physicochemical property of a liquid surface. The concept of surface tension has widespread implications in numerous natural, engineering and biomedical processes. Research to date has been largely focused on the liquid side; little attention has been paid to the vapor—the other side of the surface, despite over 100 years of study. However, the question remains as to whether the vapor plays any role, and to what extent it affects the surface tension of the liquid. Here we show a systematic study of the effect of vapor on the surface tension and in particular, a surprising observation that the vapor, not the liquid, plays a dominant role in determining the surface tension of a range of common volatile organic solutions. This is in stark contrast to results of common surfactants where the concentration in the liquid plays the major role. We further confirmed our results with a modified adsorption isotherm and molecular dynamics simulations, where highly structured, hydrogen bonded networks, and in particular a solute depletion layer just beneath the Gibbs dividing surface, were revealed.

## Introduction

Surface tension is a macroscopic, thermodynamic manifestation of molecular structure and interaction at a surface; it relates to all other physical and chemical properties. Behavior of surface tension has been implicated to widespread natural, physiological and technological processes, ranging from cloud formation, ocean wave creation and rise of sap in plants, to lung functioning, nanofabrication and nanomotor design, and controlled release of surface-to-air biological signals [1]–[6]. In many cases the surface tension of a particular system is influenced by the presence of a surface-active-agent, or surfactant. When a fresh interface is formed, the surfactant is drawn toward (or adsorbs at) the interface to achieve a thermodynamically more favorable state. The result is a reduction in free energy of the system, and in turn a decrease in surface tension.

Although surface tension has been studied extensively for over a century, particularly in the fields of colloid and surface chemistry, most research focuses on the effect of the liquid phase surfactant concentration, perhaps because of the much lower density of surfactant in the vapor phase [7]–[15]. Even though both liquid and vapor phase adsorption are examined in many physical chemistry textbooks, they are almost always considered exclusive of one another. However, when a volatile surfactant is dissolved in the liquid phase, which also exerts a finite partial pressure in the vapor phase, can adsorption from both sides of the interface still be considered independent of one another? If not, then to what extent does the vapor phase influence the interfacial properties, as compared to the liquid phase? An answer to this may well be the clue to many problems encountered in surface tension studies over the years. Previous studies have reported that aqueous alcohol solutions are particularly susceptible to errors during surface tension measurements due to solute evaporation into the vapor phase [8], [9] and although there have been numerous studies on these systems [10]–[15], the possible influence of the vapor phase on the liquid surface tension has not been considered carefully in designed experiments.

To address these questions, we present surface tension measurements from a group of slightly volatile, organic amphiphiles in aqueous solutions that illustrate the effects of both vapor and liquid phases on the surface tension. Specific attention was paid to the cases where conditions existed for communication (mass transfer) between the liquid and vapor phases. The compounds chosen for this study were 1-octanol, 1-butanol, and 1-octanoic acid. Traditionally these organic molecules are referred to as surfactants in the sense that they are surface active and tend to adsorb at an interface. However, as we will illustrate, these systems behave very differently from traditional surfactants (see Figures S1 and 2).

## Methods

### Surface Tension Measurements

Surface tension measurements were carried out using the Axisymmetric Drop Shape Analysis-Profile (ADSA-P) method [16] mainly for its high accuracy and sample environment control, see Text S1 for details. Briefly, a small pendant drop of the sample solution (drop solution) was formed inside a clear quartz cuvette above 1 ml of aqueous solution containing the same component as in the drop (environment solution, which controlled the vapor pressure). If the two liquid solutions had different surfactant concentrations, a driving force was established for molecular transfer across the vapor/liquid interface causing the surface tension of the drop solution to evolve as a result of the molecular exchange. Three distinct concentration-difference conditions were explored: positive when the drop solution concentration was greater than the environment solution concentration, negative when the drop concentration was less than the environment concentration, and zero when the two were equal.

### Molecular Dynamics Simulations

**Figure 1:**
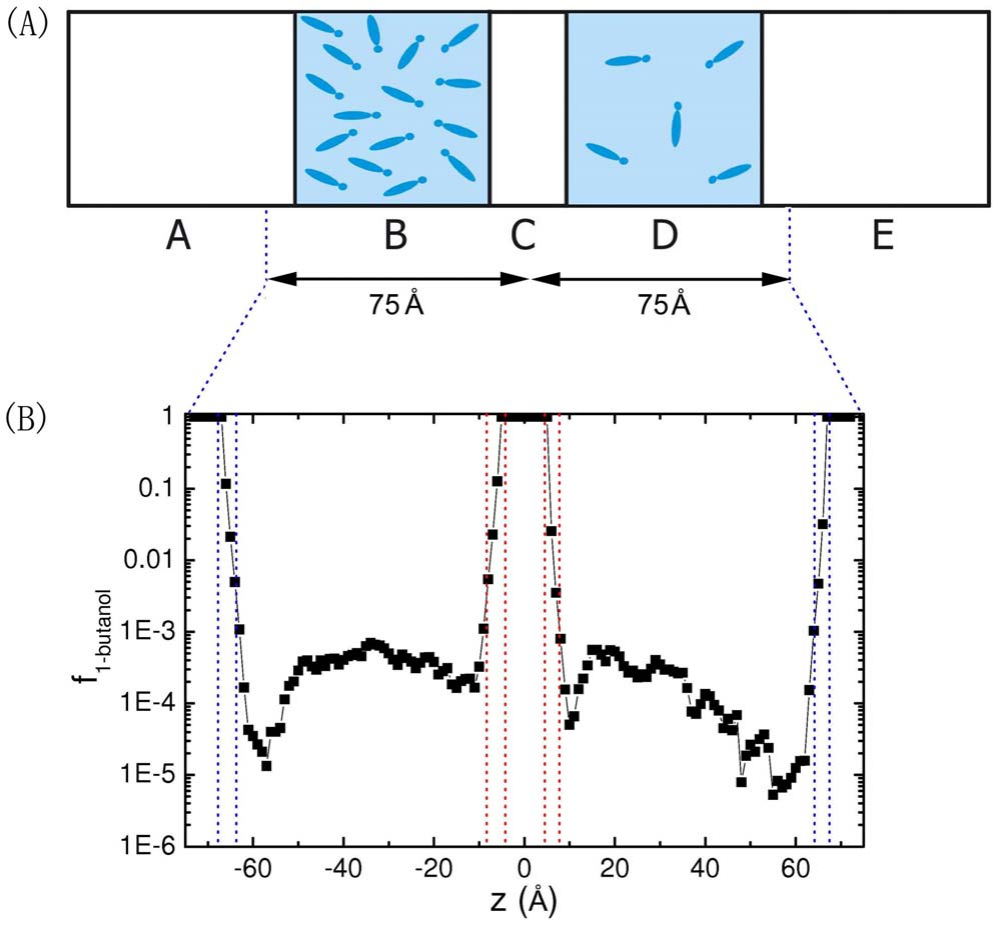
(a) Schematic of the 1-butanol in water system. Regions A, C and E are vapor phases, and regions B and D are mixtures of 1-butanol and water. Initially, 54 and 8 1-butanol molecules are in regions B and D, respectively. The z-direction dimensions of the five regions A, B, C, D and E are 84 Å, 60 Å, 12 Å, 60 Å and 84 Å, respectively. 1-butanol is allowed to evaporate to the vapor phases, while water molecules are constrained in regions B and D. (b) 1-butanol composition profile *f*_1-butanol_ as a function of its z position. The composition is defined as the number of 1-butanol molecules divided by the number of total molecules. The profile is calculated from molecular oxygen positions. To produce this profile, the system is divided into slabs of 1 Å thickness each parallel to the interfaces. As water molecules are constrained in the water boxes, *f*_1-butanol_ reaches 1 in the regions of vapor phases. The dotted lines in red and blue represent the margins of the 10–90 interfacial thickness (see Table S1).

Molecular dynamics simulations were performed using an all-atom model, with the program CHARMM [17] and the all-hydrogen parameter set PARAM22. The schematic of the simulated system is shown in Figure 1a. The whole system was 60×60×300 Å^3^, which was divided into five regions: the left vapor box (region A), the left solution box (region B), the middle vapor box (region C), the right solution box (region D) and the right vapor box (region E); four surfaces or interfaces were formed between parts A and B (interface I_AB_), B and C (interface I_BC_), C and D (interface I_CD_), D and E (interface I_DE_), respectively. The *z*-direction dimensions for the five parts A, B, C, D and E are 84 Å, 60 Å, 12 Å, 60 Å and 84 Å, respectively. This five-region system has at least two advantages: i) technically providing two-dimensional periodic boundary conditions in *x* and *y* directions, and ii) introducing two additional liquid/vapor interfaces to avoid cross-mixing of 1-butanol molecules between the two liquid phases at their outer boundaries, which would happen if the usual periodic boundary condition were employed in the *z*-direction. TIP3P water molecules were placed evenly in the two solution boxes, i.e., regions B and D; 54 and 8 1-butanol molecules were placed randomly in these two boxes, with the corresponding 1-butanol concentrations 415.3 mol/m^3^ and 61.5 mol/m^3^, respectively. Water molecules in the two solution boxes were constrained not to diffuse out of the water boxes using a harmonic potential with the amplitude 50.0 kcal/mol; thus the viscosity effect was neglected in the vapor phases, and 1-butanol molecules can transfer relatively free between the two interfaces through the vapor phase C. As a result, the exchange rate of 1-butanol molecules was facilitated, which resulted in a reduced demand of the vast of computer resources. No other constraints were applied. The two inner interfaces are responsible for 1-butanol molecule exchange between the two liquid solution boxes. The reference surface tension utilized in the simulations was 45.0 mN/m, considering the difference between the simulated value and the experimental value in relation to the force-field effects [18]. The simulation temperature was controlled at 330 K in order to accelerate the 1-butanol exchange. The volume of the whole system was kept constant during the simulation. The total simulation time for each run was 6 ns, and the last 3 ns data were used for analysis. In the simulations, a 12 Å effective nonbonded cutoff distance was used, and at the periodic boundary, the image atoms within 14 Å from the atoms in the primary box were considered [17]. The focus of the simulations was on the 1-butanol molecule exchange or transfer between the two inner interfaces.

## Results and Discussion

The time-dependent or Dynamic Surface Tension (DST) profiles for the aqueous 1-butanol system are shown in Figure S2. Each profile begins with an initial induction period approximately 10 to 100 seconds in length, followed by an increase or decrease in surface tension toward a final steady-state or “equilibrium”. In each case, the overall trend of the surface tension is controlled by the concentration difference between the drop and the environment solution. Under the positive concentration-difference condition, i.e., the drop solution concentration being greater than the environment solution concentration, the surface tension increases after the induction period. Under the negative concentration-difference condition, the surface tension decreases after the induction period. Under the zero concentration-difference condition, the surface tension remains essentially constant as there is no driving force for molecular transfer. Interestingly, for a given environment solution concentration, almost the same final surface tension value is attained by each profile regardless of the concentration of the drop solution. In each case the final constant surface tension is close to the surface tension of the environment solution. These results suggest that initially the surface tension is controlled by a combination of the liquid and vapor phase concentrations, whereas at the final steady-state the surface tension is determined primarily by the vapor phase.

To determine if the results were limited to the 1-butanol system, aqueous solutions of 1-octanol and 1-octanoic acid were also investigated. The DST results from the 1-octanol and 1-octanoic acid solutions (see Figures S3 and S4, and other published work [19]) show essentially the same trends when compared to the results from 1-butanol. This suggests that the observed phenomenon is rather general and shared by this class of common organics.

To understand such surface tension behavior, especially at the initial and final steady-state, we required an appropriate adsorption isotherm to model our experimental results. The purpose of an adsorption isotherm is to relate the surfactant concentration in the bulk and the amount adsorbed at the interface [15], [20]. There were no isotherms that take into account adsorption from both the liquid and vapor phase simultaneously. Thus, we derived a modified adsorption isotherm based on the same rationales as those of the classic Langmuir isotherm [20] while incorporating adsorption/desorption from both sides of the vapor/liquid interface; specifically at the Gibbs dividing surface (see below and [19]),
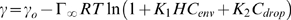
(1)where γ and γ_o_ are the surface tensions of the solution and the pure solvent, Γ_∞_ the maximum surface concentration of surfactant, R the universal gas constant, T the temperature, K_1_ and K_2_ the equilibrium constants for adsorption from the vapor and liquid phases, H the Henry's law constant of surfactant, C_env_ the concentration in the environment solution, and C_drop_ the concentration in the drop solution.

The equilibrium parameters K_1_, K_2_, and Γ_∞_, were determined by fitting Equation (1) to experimental data through nonlinear regression [21]. The parameters, generated using data collected from initial and final steady-state conditions, are listed in Table 1. The modified isotherm fits experimental data quite well (see Figure S5).

**Table 1:** Fitting parameters for Equation (1).

Surfactant	Fitting	Γ_∞_ (mol/m^2^)	K_1_ a (m^3^/mol)	K_2_ (m^3^/mol)
Butanol	Initial	5.91×10^−6^	0.0063	0.0205
	Final	5.95×10^−6^	0.0216	0.0007

a The values of K_1_ include the Henry's law constant so that the units are uniform with K_2_.

From Table 1 one can see that initially both the liquid and the vapor phase contribute to adsorption at the interface as illustrated by the comparable values of K_1_ and K_2_. At final steady-state, adsorption from the vapor phase represents the major contribution as reflected by the difference in the magnitudes of K_1_ and K_2_ (K_2_ is only 3.2% of K_1_). The results support the experimental observations that initially the surface tension is determined by a combination of adsorption from the liquid and the vapor phase, whereas at the final steady-state the surface tension is determined primarily by adsorption from the vapor phase.

At the final steady-state or experimental “equilibrium” the surface tension reaches a final, constant value which seems to be related only to the vapor phase surfactant concentration. This leads us to speculate that a significant energy barrier may have been forged on the liquid side of the interface, causing the molecular exchange between the liquid phase and the interface to be severely diminished. It should be noted that even under the steady-state conditions the concentration difference between the drop solution and the environment solution is maintained (see Figure S6).

To support the analyses above, molecular dynamics simulations based on an all-atom model were performed for the 1-butanol system. The simulation system contains four liquid-vapor interfaces, and the two inner interfaces are of interest, responsible for the 1-butanol exchange/transfer between regions B and D, Figure 1a.

Figure 1b shows that all interfaces are populated by 1-butanol molecules. Also observed is a 1-butanol depleted region, or depletion layer, just beneath each surface, defined by the Gibbs dividing surface. To obtain the location of the Gibbs dividing surface and thickness of each interface, the density profile of the mixture of 1-butanol and water was fitted with a hyperbolic tangent functional form [22], [23], see Table S1. In the simulations, all 1-butanol molecules transferred across the five regions. For example, in the final 2 ns simulation trajectory, the average time for a single 1-butanol molecule to transfer from region B to interface I_BC_, interface I_BC_ to region C, region C to interface I_CD_, interface I_CD_ to region D, region D to interface I_CD_, interface I_CD_ to region C, region C to interface I_BC_, and interface I_BC_ to region B is 85, 40, 391, 98, 77, 59, 897 and 85 ps, respectively.

**Figure 2:**
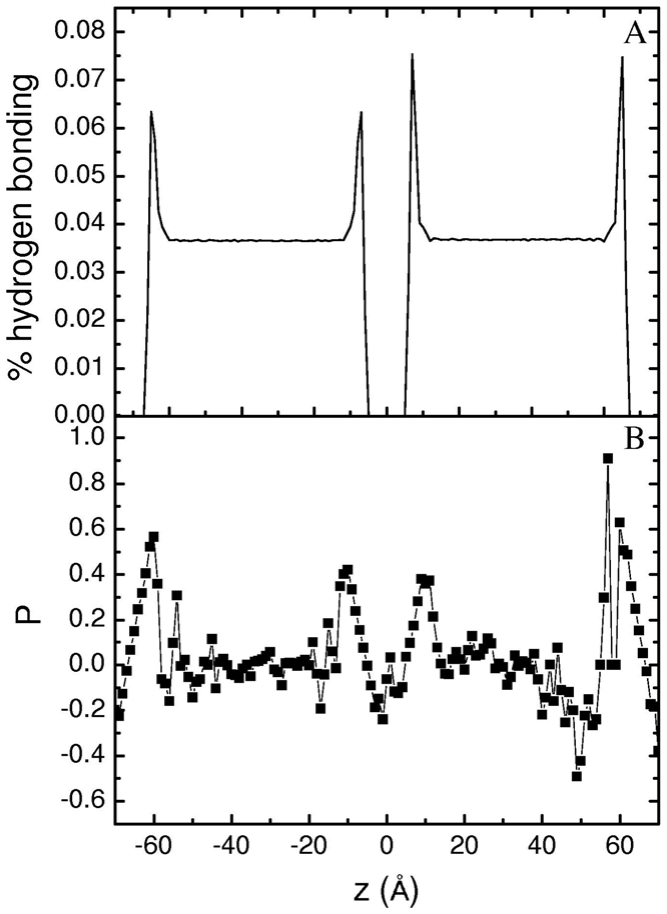
(a) The degree of hydrogen bonding as a function of the *z* coordinate, which is defined as the number of 1-butanol – 1-butanol, 1-butanol – water and water – water hydrogen bonds divided by the number of molecules in the first solvation shell. (b) Orientational order parameter *P* profile for 1-butanol molecules as a function of its z location.

To determine the molecular configuration/structure at the interface, the number density profiles for the hydroxyl hydrogen, hydroxyl oxygen, methyl carbon, water hydrogen, and water oxygen were computed (see Figure S7). These profiles indicate that the outermost region of each surface, i.e., the region closest to the vapor phase, is populated by the 1-butanol hydrophobic tails, while hydrophilic headgroups (hydroxyl groups) are about 3 Å toward the interior of the bulk liquid. This is consistent with the fact that alcohol molecules are amphiphilic or surface active. To gain more structural information, the extent/degree of hydrogen bonding was evaluated by the average number of all hydrogen bonds divided by the average number of molecules in the first solvation shell, defined by the first minimum of the oxygen-oxygen radial distribution function (RDF) [23]. The degree of hydrogen bonding increases at the interface as shown in Figure 2a.

The orientational preference of 1-butanol molecules at the liquid/vapor interface was also examined by an orientational order parameter defined as
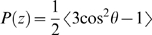
(2)where *θ* is the angle between the surface normal and the molecule internal vector, which is defined as the line connecting the first and the third carbon atoms of the 1-butanol molecule. For an isotropic system, *P* is near zero; P = −1/2 indicates that all of the vectors in the system are parallel to the interface; P =  1 indicates all of the vectors in the system are perpendicular to the interface. The results in Figure 2b indicate that the 1-butanol molecules in the bulk are almost isotropic, but prefer to stand upright near/at the surface, showing a strong surface ordering. The change in local structures of 1-butanol and water molecules from the bulk region to the interface was in addition examined in terms of the oxygen-hydrogen RDFs (see Figure S8). While positions of the first peaks were similar, the height of the first peak increased from the bulk region to the interface, indicating that more hydrogen bonds form at the interface [23].

The results of hydrogen bonding and amphiphilic orientation, along with those of density profiles, imply that a local clustering or ordering of 1-butanol molecules may occur at the interface. This local structuring could be a hydrogen bonded network [24], [25]. Coupling with the 1-butanol depletion layer, it is conceivable that a “barrier” is formed against transport/exchange of 1-butanol between the bulk liquid and the interface, thereby supporting the notion of a high energy barrier postulated above. On the other hand, the exchange of 1-butanol molecules between the vapor phase and the interface is relatively free; thus the vapor pressure becomes the dominant factor affecting adsorption at the surface, impacting surface concentration and surface tension.

### Conclusions

We have shown that the vapor phase, or adsorption from the vapor phase, represents a significant dynamic affecting the aqueous surface tension of this class of rather common volatile organic molecules, particularly at the final steady-state or “equilibrium” where it seems to be the primary factor. The results are in contrast to traditional surfactants where the surface tension is mainly controlled by liquid phase adsorption. For the current systems, adsorption from both the liquid and the vapor phase must be considered mutually if the objective is to examine the complete adsorption process. The modified adsorption isotherm can be used to describe steady-state surface tension data for this class of molecules, under conditions where conventional equations do not apply. It is particularly useful at initial steady-state conditions when the surface tension is influenced by both liquid and vapor phase surfactant concentrations. It is important in all surface tension related applications to understand that aqueous solutions of these compounds behave very differently from traditional surfactants. We further confirmed this finding with extensive molecular dynamics simulations, where highly structured, hydrogen bonded networks, and in particular an organic depletion layer, were revealed near the vapor-liquid interface. We suspect that the volatile, organic compounds considered in the current study may represent a rather general group of molecules whose surface behavior is unique to that of conventional, non-volatile, or even many volatile, surfactants.

## Supporting Information

Text S1 Supporting information text
(0.07 MB DOC)

Table S1 Supporting information table
(0.03 MB DOC)

Figure S1 Dynamic surface tension profile of some traditional surfactant systems. Environment solution is pure water for both cases. (A) Aqueous solutions of octaethylene glycol monododecyl ether (C12E8) at drop concentrations of 0.008 mol/m3 (◊), 0.04 mol/m3 (□), 0.093 mol/m3 (Δ). (B) Aqueous solutions of Igepal CO-720 at drop concentrations of 0.00123 mol/m3 (◊), 0.00657 mol/m3 (○), 0.00985 mol/m3 (□), 0.0246 mol/m3 (Δ).
(0.13 MB DOC)

Figure S2 Aqueous 1-butanol dynamic surface tension profiles for drop solution concentrations of 20 mol/m3 (◊), 60 mol/m3 (□), 100 mol/m3 (Δ), and 400 mol/m3 (○). Each graph represents a different environment solution concentration: (a) pure water, (b) 60 mol/m3, (c) 100 mol/m3, and (d) 400 mol/m3. Note that the data shown here are somewhat similar to those of Prpich AM, Biswas ME, Chen P [(2008) “Adsorption kinetics of aqueous n-alcohols: a new kinetic equation for surfactant transfer,” J. Phys. Chem. C 112: 2522–2528], but with new concentration combinations added.
(0.45 MB DOC)

Figure S3 Aqueous 1-octanol dynamic surface tension profiles for drop solution concentrations of 0.2 mol/m3 (◊), 0.4 mol/m3 (▪), 0.6 mol/m3 (Δ), 0.8 mol/m3 (•), 1.0 mol/m3 (□), and 2.92 mol/m3 (♦). Each graph represents a different environment solution concentration; (A) pure water, (B) 0.6 mol/m3, (C) 1.0 mol/m3, and (D) 2.92 mol/m3.
(0.35 MB DOC)

Figure S4 Aqueous 1-octanoic acid dynamic surface tension profiles for drop solution concentrations of 0.2 mol/m3 (◊), 0.5 mol/m3 (□), 0.8 mol/m3 (Δ), and 2.0 mol/m3 (○). Each graph represents a different environment solution concentration; (A) Pure water, (B) 0.8 mol/m3, and (C) 2.0 mol/m3.
(0.25 MB DOC)

Figure S5 (A) Initial steady-state surface tension of aqueous 1-butanol as a function of drop solution concentration for Cenv  = 0 mol/m3 (Δ), 60 mol/m3 (□), 100 mol/m3 (Δ), and 400 mol/m3 (○). Note that (A) is a repeat plot of Figure 1 of Prpich AM, Biswas ME, Chen P [(2008) “Adsorption kinetics of aqueous n-alcohols: a new kinetic equation for surfactant transfer,” J. Phys. Chem. C 112: 2522–2528] and included here for comparison purposes. (B) Final steady-state surface tension of aqueous 1-butanol as a function of environment solution concentration for Cdrop  = 20 mol/m3 (Δ), 60 mol/m3 (□), 100 mol/m3 (Δ), and 400 mol/m3 (○). (C) Initial steady-state surface tension of aqueous 1-octanol as a function of drop solution concentration for Cenv  = 0 mol/m3 (Δ), 0.2 mol/m3 (◊), 0.6 mol/m3 (Δ), 0.8 mol/m3 (○), 1.0 mol/m3 (□), and 2.92 mol/m3 (◊). (D) Final steady-state surface tension of aqueous 1-octanol as a function of environment solution concentration for Cdrop  = 0.2 mol/m3 (◊), 0.4 mol/m3 (□), 0.6 mol/m3 (Δ), 0.8 mol/m3 (○), 1.0 mol/m3 (□), and 2.92 mol/m3 (◊). Solid lines represent theoretical predictions from Equation (1).
(0.35 MB DOC)

Figure S6 Aqueous 1-octanol dynamic surface tension profiles for consecutive drops from a continuous run using the same syringe and environment solution; Drop #1 (□), Drop #2 (◊). Drop solution concentration is 1.0 mol/m3 with pure water as the environment solution.
(0.03 MB DOC)

Figure S7 (A) Number density profiles of 1-butanol molecules. The values for hydroxyl hydrogen, hydroxyl oxygen and methyl carbon are depicted as black squares, red circles and green triangles, respectively. (B) Number density profiles of water molecules. The profiles connected using the black dotted line and the red solid line are for water hydrogen and oxygen, respectively.
(0.60 MB DOC)

Figure S8 Hydroxyl oxygen - water hydrogen (a) and water oxygen - water hydrogen (b) radial distribution functions. The black, red and blue curves correspond to the molecules located at the Gibbs dividing surface (z = −6.27 Å), 4 Å (z = −10.27 Å) and 8 Å (z = −20.27 Å) away from the dividing surface into the liquid. Note that the hydrogen bonding H…O distance is typically 1.6∼2.0 Å, and the distance of the first peak in RDF is well within the hydrogen bonding distance.
(0.33 MB DOC)
