# Supplementary material for: Tension at the Surface: Which Phase Is More Important, Liquid or Vapor?
Source: PLoS One. 2009 Dec 14;4(12):e8281. doi: 10.1371/journal.pone.0008281 (PMC2788621; doi:10.1371/journal.pone.0008281)
Supplement: Text S1 — Supporting information text (0.07 MB DOC) [file pone.0008281.s001.doc]

# Supporting Text S1

# Materials and Methods

## Materials

The chemicals for this study (1-octanol, 1-butanol, and 1-octanoic acid) were purchased from Sigma-Aldrich Canada (Oakville, Ontario, Canada). Estimated water solubility [1, 2] and vapor pressure data [3-5] are 4.15 mol/m^3^ and 0.08 mmHg (25 ℃) for 1-cotanol, 1060 mol/m^3^ and 5.02 mmHg (20 ℃) for 1-butanol, and 4.72 mol/m^3^ and 0.008 mmHg (25 ℃) for 1-octanoic acid. The purity of all chemicals was greater than 99% and no further purification was performed before use. The water used for the solution preparation was purified by an Ultra-Pure Water System from Millipore Co. The resistivity of the water after purification was measured to be at least 18.2 MΩ and the surface tension was 72.5 ± 0.5 mN/m. Samples were tested shortly after preparation and stored for no longer than two weeks. New samples were prepared as necessary.

## Surface Tension Measurements

The surface tension measurements were performed using the Axisymmetric Drop Shape Analysis-Profile (ADSA-P) method. Details of this technique have been published previously [6-8]. For each experiment, a pendant drop of the sample under test (referred to as the drop solution) was formed inside the quartz cuvette above 1 ml of aqueous solution (referred to as the environment solution) containing the same component as the drop. The environment solution was added to facilitate adsorption from the vapor side of the interface by creating a surfactant vapor phase surrounding the drop solution. If the two liquid solutions had different surfactant concentrations, a driving force was established for molecular transfer across the vapor/liquid interface causing the surface tension of the drop solution to evolve as a result of the exchange. Before each set of experiments, the gas-tight syringe was cleaned by ultrasonic and repeated rinsing with purified water. After cleaning, approximately 0.3-0.4 ml of aqueous solution was drawn into the syringe. The environment solution was added to the cuvette using a 1 ml micropipette. The syringe was then fed into the cuvette and the chamber was sealed. The system was allowed to equilibrate for 15 minutes after which, the drop was formed using the motorized syringe. The temperature of the chamber was controlled by a water cooling bath. For the 1-octanol and 1-octanoic acid solutions the temperature was maintained at 20 °C and for the 1-butanol solutions the temperature was increased to 25 °C. Image capture began immediately after the drop was formed and continued until the surface tension of the drop was no longer changing (~2-5 hours). The time-dependent or Dynamic Surface Tension (DST) profiles of some traditional surfactant systems were measured and are shown in Figure S1. For both cases, the surrounding environment is saturated with water vapor. The primary distinction between these results and the results of the volatile, organic amphiphiles investigated in this study is the surface tension of traditional surfactant solutions, especially at equilibrium, is mainly a function of the concentration of the liquid solution and independent of the vapor phase. Results of DST for 1-butanol, 1-octanol and 1-octanoic acid aqueous solutions are shown in Figures S2-4.

**Fitting of Equation (1) to the Surface Tension Data**

The equilibrium parameters K_1_, K_2_, and Γ_∞_, can be determined for any system by fitting Equation (1) of the text to experimental data through nonlinear regression [9]. For this study the parameters were evaluated for the aqueous 1-octanol system using data from a central composite design (CCD) of experiment, and for the aqueous 1-butanol system using existing experimental data. The optimization routine was implemented in MATLAB for both initial and final (equilibrium) surface tension data.

In this analysis the effect of surface curvature on the vapor pressure of the drop solution has been neglected. According to the Kelvin equation the vapor pressure around a curved surface is greater than that of a similar flat surface. However, for droplets with radius larger than 10^-4^ cm the effect is essentially negligible [10]. In our experiments the radius of curvature of the drop solution is on the order of 10^-1^ cm. The equilibrium parameters K_1_, K_2_, and Γ_∞_, generated using data collected from initial and final steady-state surface tension conditions for 1-butanol system, are listed in Table 1 of the main text. The corresponding parameters for 1-octanol system are: for initial steady state, Γ_∞_ = 8.19x10^-6^ mol/m^2^, K_1_ = 0.6440 m^3^/mol, K_2_ = 1.4316 m^3^/mol; for “equilibrium” state, Γ_∞_ = 7.87x10^-6^ mol/m^2^, K_1_ = 1.9188 m^3^/mol, K_2_ = 0.0887 m^3^/mol. It should be noted that the values of K_1_ included the Henry’s law constant so that the units are consistent with K_2_. In all cases, the modified isotherm fits the experimental data quite well, as shown in Figure S5.

For both surfactants the values obtained for the maximum surface concentration (Γ_∞_) are very similar for the initial and final steady-state fittings and compare well with published values [11-13]. This parameter is a function of the physical size of a given molecule at the surface and thus, should be constant. Conversely, the adsorption equilibrium constants, K_1_ and K_2_, differ considerably between the initial and final phase fittings for a given surfactant. Also, the value of K_1_ is often quite different than the value of K_2_ in any given case. These parameters are often used as a gauge of the surface activity or efficiency of a surfactant [14]. A large value implies the surfactant is more surface active or conducive to adsorption and thus, more efficient at reducing the surface tension. Therefore, the value of K_1_ is indicative of the contribution to adsorption from the vapor phase and K_2_ the contribution from the liquid phase. For a given surfactant the difference in the values of K_1_ and K_2_ between the two fittings illustrates the difference in adsorption at initial and final steady-state conditions.

Examining the adsorption equilibrium constants mentioned above for any given surfactant one can see that initially both the liquid and the vapor phase contribute to adsorption at the interface as illustrated by the comparable values of K_1_ and K_2_. At final steady-state conditions, adsorption from the vapor phase represents the major contribution as reflected by the difference in the magnitudes of K_1_ and K_2_ (K_2_ is only 4.6% of K_1_ for 1-octanol, and 3.2% for 1-butanol). The results support the experimental observations discussed previously that initially the surface tension is determined by a combination of adsorption from the liquid and the vapor phase, whereas at the final steady-state the surface tension is determined primarily by adsorption from the surrounding vapor.

At the final steady-state or experimental “equilibrium” the surface tension reaches a final, constant value which seems to be directly related to the vapor phase surfactant concentration. This leads us to speculate that a significant energy barrier may have been forged on the liquid side of the interface, causing the molecular exchange between the liquid phase and the interface to be severely diminished (see also the text).

To support this hypothesis further we need to show that even at steady-state conditions the concentration difference between the drop solution and the environment solution is maintained. Thus, the surface tension of a 1-octanol solution was measured for consecutive drops from a continuous run using the same syringe and environment solution (see Figure S6). In this experiment, the surface tension of the first drop was measured according to the normal procedure. At the end of the run, the drop was discharged into the environment, a second drop was formed without removing the syringe from the chamber, and the surface tension was recorded. The profiles from the two consecutive runs are nearly identical suggesting that, even at the final steady-state of the first drop, the concentration difference between the drop solution and the environment solution is maintained; otherwise, the surface tension of the second drop would be constant over the course with a zero concentration difference. This suggests that even though the liquid and the vapor reach a steady-state, a true thermodynamic equilibrium, where the chemical potential and hence the concentration of species in all phases is equal, has not been attained.

**Fitting for the Gibbs Dividing Surface**

The density profile across an interface can be fitted with an analytical function, such as the hyperbolic tangent or Fermi type function [15, 16]. The functional form chosen is as follow:

$\rho(z)=\frac{1}{2}(\rho_{l}+\rho_{v})-\frac{1}{2}(\rho_{l}-\rho_{v})\tanh[(z-z_{0})/2d]$ (S1)

where *ρ_l_* and *ρ_v_* are the liquid and vapor densities, respectively, *z_0_* is the position of the middle of the transition layer, i.e., the location of the Gibbs dividing surface, and *d* is the parameter for the thickness of the interface. The usual “10-90 thickness” of the Gibbs dividing surface *t* is related to *d* as in *t* = 4.394 *d* [15]. The densities of the 1-butanol and water mixture *ρ(z)* are evaluated using fluid slabs of 1 Å thickness parallel to the interface. Four distinct liquid-vapor interfaces are shown in Table S1. It is also found that the thickness of the interface at higher 1-butanol concentrations is larger than that at lower 1-butanol concentrations. The density profiles in Figure S7 show that at the interface the 1-butanol density increases significantly, while the water density reduces, suggesting that there may be a local clustering of 1-butanol molecules at the interface, consistent with experimental findings that alcohol molecules in the surface prefer to stay within the vicinity of each other [17-19].

# References

1. Abraham MH, Le J (1999) The correlation and prediction of the solubility of compounds in water using an amended solvation energy relationship. J. Pharm. Sci. 88:868-880.

2. Yaws CL et al. (1998) Solubility and Henry's law constants for alcohols in water. Waste Management 17:541-547.

3. Nasirzadeh K, Neueder R, Kunz W (2006) Vapor pressure determination of the aliphatic C5 to C8 1-alcohols. J. Chem. Eng. Data 51:7-10.

4. Munday EB, Mullins JC, Edie DD (1980) Vapor pressure data for toluene, 1-pentanol, 1-butanol, water, and 1-propanol and for the water and 1-propanol system from 273.15 to 323.15 K. J. Chem. Eng. Data 25:191-194.

5. Ambrose D, Ghiassee NB (1987) Vapor pressures and critical temperatures and critical pressures of some alkanoic acids: C1 to C10. The Journal of Chemical Thermodynamics 19:505-517.

6. Lahooti S, del Rio OI, Neumann AW, Cheng P (1996) in Applied Surface Thermodynamics. eds. Neumann AW, Spelt JK (Marcel Dekker, New York), pp 441-508.

7. Rotenberg Y, Boruvka L, Neumann AW (1983) Determination of surface tension and contact angle from the shapes of axisymmetric fluid interfaces. J. Colloid Interface Sci. 93:169-183.

8. del Rio OI, Neumann AW (1997) Axisymmetric drop shape analysis: Computational methods for the measurement of interfacial properties from the shape and dimensions of pendant and sessile drops. J. Colloid Interface Sci. 196:136-147.

9. Montgomery DC (2001) Design and Analysis of Experiments. 5 ed. (John Wiley & Sons, New York).

10. MacLeod CA, Radke CJ (1994) Surfactant exchange kinetics at the air-water-interface from the dynamic tension of growing liquid-drops. J. Colloid Interface Sci. 166:73-88.

11. Lin SY, Wang WJ, Hsu CT (1997) Adsorption kinetics of 1-octanol at the air-water interface. Langmuir 13:6211-6218.

12. Miller R, Lunkenheimer K (1986) Adsorption kinetics measurements of some nonionic surfactants. Colloid Polym. Sci. 264:357-361.

13. Wu N, Parris J (2000) Interaction of water-soluble acrylic polymers with alcohols in aqueous solutions. Colloids and Surfaces A: Physicochemical and Engineering Aspects 167:179-187.

14. Chang CH, Franses EI (1995) Adsorption dynamics of surfactants at the air/water interface: a critical review of mathematical models, data, and mechanisms. Colloids and Surfaces A: Physicochemical and Engineering Aspects 100:1-45.

15. Matsumoto M, Kataoka Y (1988) Study on liquid-vapor interface of water. І. Simulation results of thermodynamic properties and orientational structure. J. Chem. Phys. 88:3233-3245.

16. Chang TM, Dang LX (2005) Liquid-vapor interface of methanol-water mixtures: A molecular dynamics study. J. Phys. Chem. B 109:5759-5765.

17. Gilányi T, Mészáros R, Varga I (2000) Phase transition in the adsorbed layer of catanionic surfactants at the air/solution interface. Langmuir 16, 3200-3205.

18. Varga I et al. (2005) Observation of a liquid-gas phase transition in monolayers of alkyltrimethylammonium alkyl sulfates adsorbed at the air/water interface. J. Phyc. Chem. B 109:872-878.

19. Pártay LB, Jedlovszky P, Vincze A, Horvai G (2008) Properties of free surface of water-methanol mixtures. Analysis of the truly interfacial molecular layer in computer simulation. J. Phys. Chem. B 112:5428-5438.
