# Supplementary material for: Tension at the Surface: Which Phase Is More Important, Liquid or Vapor?
Source: PLoS One. 2009 Dec 14;4(12):e8281. doi: 10.1371/journal.pone.0008281 (PMC2788621; doi:10.1371/journal.pone.0008281)
Supplement: Table S1 — Supporting information table (0.03 MB DOC) [file pone.0008281.s002.doc]

**Table S1.** Location of the Gibbs dividing surface and the 10-90 thickness of interfaces.

| Interface between parts | Z_Gibbs_(Å) | Thickness (Å) |
| --- | --- | --- |
| A-B | -65.70 ± 0.02 | 2.04 ± 0.07 |
| B-C | -6.27 ± 0.02 | 2.07 ± 0.07 |
| C-D | 6.15 ± 0.02 | 1.60 ± 0.06 |
| D-E | 65.83 ± 0.02 | 1.63 ± 0.06 |
