# Supplementary material for: Tension at the Surface: Which Phase Is More Important, Liquid or Vapor?
Source: PLoS One. 2009 Dec 14;4(12):e8281. doi: 10.1371/journal.pone.0008281 (PMC2788621; doi:10.1371/journal.pone.0008281)
Supplement: Figure S1 — Dynamic surface tension profile of some traditional surfactant systems. Environment solution is pure water for both cases. (A) Aqueous solutions of octaethylene glycol monododecyl ether (C12E8) at drop concentrations of 0.008 mol/m3 (◊), 0.04 mol/m3 (□), 0.093 mol/m3 (Δ). (B) Aqueous solutions of Igepal CO-720 at drop concentrations of 0.00123 mol/m3 (◊), 0.00657 mol/m3 (○), 0.00985 mol/m3 (□), 0.0246 mol/m3 (Δ). (0.13 MB DOC) [file pone.0008281.s003.doc]

**A**

**B**

**Figure S1.** Dynamic surface tension profile of some traditional surfactant systems. Environment solution is pure water for both cases. (A) Aqueous solutions of octaethylene glycol monododecyl ether (C_12_E_8_) at drop concentrations of 0.008 mol/m^3^ (◊), 0.04 mol/m^3^ (□), 0.093 mol/m^3^ (Δ). (B) Aqueous solutions of Igepal CO-720 at drop concentrations of 0.00123 mol/m^3^ (◊), 0.00657 mol/m^3^ (○), 0.00985 mol/m^3^ (□), 0.0246 mol/m^3^ (Δ).
