# Supplementary material for: Tension at the Surface: Which Phase Is More Important, Liquid or Vapor?
Source: PLoS One. 2009 Dec 14;4(12):e8281. doi: 10.1371/journal.pone.0008281 (PMC2788621; doi:10.1371/journal.pone.0008281)
Supplement: Figure S2 — Aqueous 1-butanol dynamic surface tension profiles for drop solution concentrations of 20 mol/m3 (◊), 60 mol/m3 (□), 100 mol/m3 (Δ), and 400 mol/m3 (○). Each graph represents a different environment solution concentration: (a) pure water, (b) 60 mol/m3, (c) 100 mol/m3, and (d) 400 mol/m3. Note that the data shown here are somewhat similar to those of Prpich AM, Biswas ME, Chen P [(2008) “Adsorption kinetics of aqueous n-alcohols: a new kinetic equation for surfactant transfer,” J. Phys. Chem. C 112: 2522–2528], but with new concentration combinations added. (0.45 MB DOC) [file pone.0008281.s004.doc]

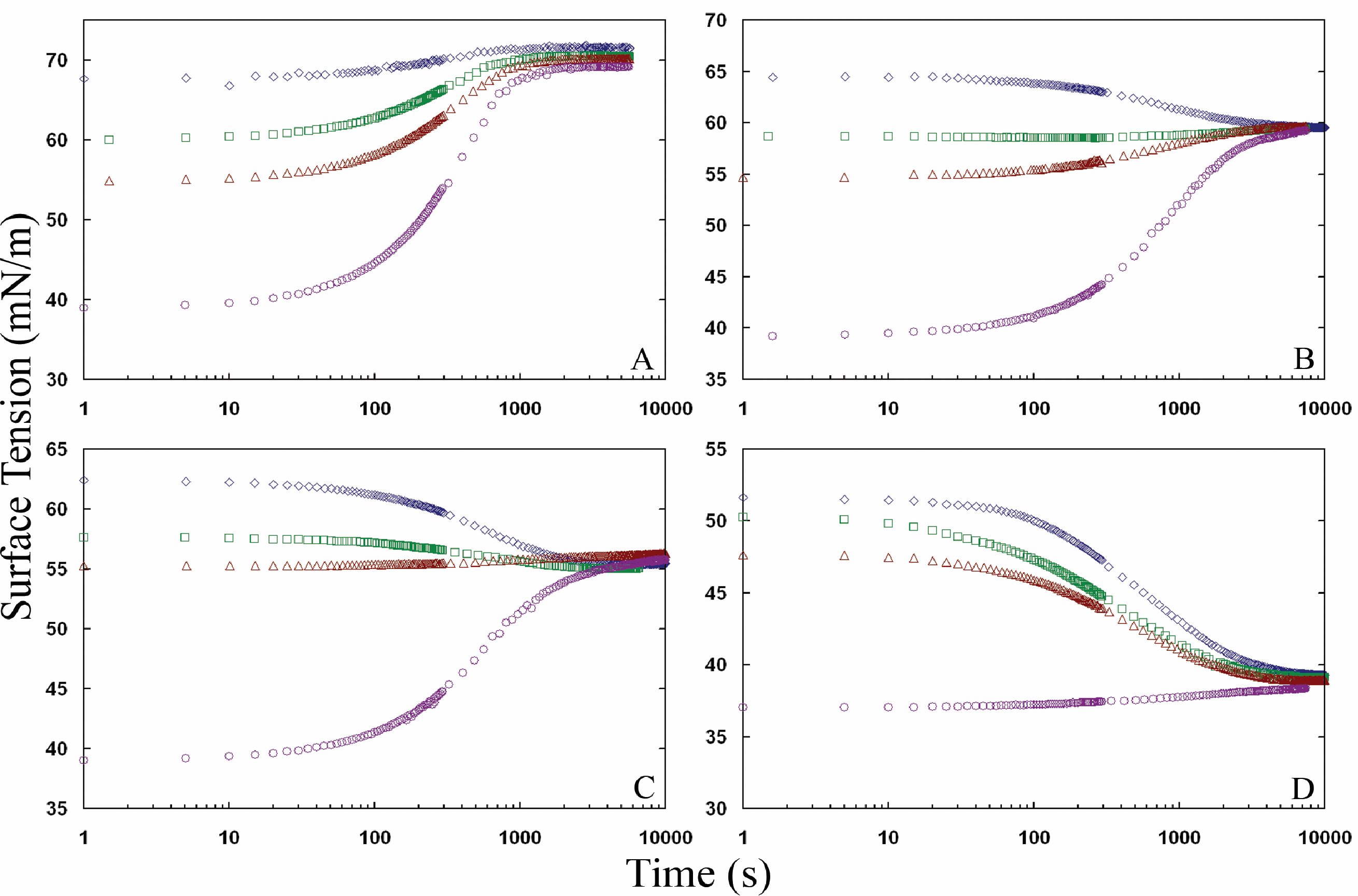


**Figure S2:** Aqueous 1-butanol dynamic surface tension profiles for drop solution concentrations of 20 mol/m^3^ (◊), 60 mol/m^3^ (□), 100 mol/m^3^ (∆), and 400 mol/m^3^ (○). Each graph represents a different environment solution concentration: (a) pure water, (b) 60 mol/m^3^, (c) 100 mol/m^3^, and (d) 400 mol/m^3^. Note that the data shown here are somewhat similar to those of Prpich AM, Biswas ME, Chen P [(2008) “Adsorption kinetics of aqueous n-alcohols: a new kinetic equation for surfactant transfer,” J. Phys. Chem. C 112: 2522-2528], but with new concentration combinations added.
