# Supplementary material for: Tension at the Surface: Which Phase Is More Important, Liquid or Vapor?
Source: PLoS One. 2009 Dec 14;4(12):e8281. doi: 10.1371/journal.pone.0008281 (PMC2788621; doi:10.1371/journal.pone.0008281)
Supplement: Figure S3 — Aqueous 1-octanol dynamic surface tension profiles for drop solution concentrations of 0.2 mol/m3 (◊), 0.4 mol/m3 (▪), 0.6 mol/m3 (Δ), 0.8 mol/m3 (•), 1.0 mol/m3 (□), and 2.92 mol/m3 (♦). Each graph represents a different environment solution concentration; (A) pure water, (B) 0.6 mol/m3, (C) 1.0 mol/m3, and (D) 2.92 mol/m3. (0.35 MB DOC) [file pone.0008281.s005.doc]

**Figure S3.** Aqueous 1-octanol dynamic surface tension profiles for drop solution concentrations of 0.2 mol/m^3^ (◊), 0.4 mol/m^3^ (■), 0.6 mol/m^3^ (∆), 0.8 mol/m^3^ (●), 1.0 mol/m^3^ (□), and 2.92 mol/m^3^ (♦). Each graph represents a different environment solution concentration; (A) pure water, (B) 0.6 mol/m^3^, (C) 1.0 mol/m^3^, and (D) 2.92 mol/m^3^.
