# Supplementary material for: Tension at the Surface: Which Phase Is More Important, Liquid or Vapor?
Source: PLoS One. 2009 Dec 14;4(12):e8281. doi: 10.1371/journal.pone.0008281 (PMC2788621; doi:10.1371/journal.pone.0008281)
Supplement: Figure S4 — Aqueous 1-octanoic acid dynamic surface tension profiles for drop solution concentrations of 0.2 mol/m3 (◊), 0.5 mol/m3 (□), 0.8 mol/m3 (Δ), and 2.0 mol/m3 (○). Each graph represents a different environment solution concentration; (A) Pure water, (B) 0.8 mol/m3, and (C) 2.0 mol/m3. (0.25 MB DOC) [file pone.0008281.s006.doc]

**Figure S4.** Aqueous 1-octanoic acid dynamic surface tension profiles for drop solution concentrations of 0.2 mol/m^3^ (◊), 0.5 mol/m^3^ (□), 0.8 mol/m^3^ (Δ), and 2.0 mol/m^3^ (○). Each graph represents a different environment solution concentration; (A) Pure water, (B) 0.8 mol/m^3^, and (C) 2.0 mol/m^3^.
