# Supplementary material for: Tension at the Surface: Which Phase Is More Important, Liquid or Vapor?
Source: PLoS One. 2009 Dec 14;4(12):e8281. doi: 10.1371/journal.pone.0008281 (PMC2788621; doi:10.1371/journal.pone.0008281)
Supplement: Figure S5 — (A) Initial steady-state surface tension of aqueous 1-butanol as a function of drop solution concentration for Cenv = 0 mol/m3 (Δ), 60 mol/m3 (□), 100 mol/m3 (Δ), and 400 mol/m3 (○). Note that (A) is a repeat plot of Figure 1 of Prpich AM, Biswas ME, Chen P [(2008) “Adsorption kinetics of aqueous n-alcohols: a new kinetic equation for surfactant transfer,” J. Phys. Chem. C 112: 2522–2528] and included here for comparison purposes. (B) Final steady-state surface tension of aqueous 1-butanol as a function of environment solution concentration for Cdrop = 20 mol/m3 (Δ), 60 mol/m3 (□), 100 mol/m3 (Δ), and 400 mol/m3 (○). (C) Initial steady-state surface tension of aqueous 1-octanol as a function of drop solution concentration for Cenv = 0 mol/m3 (Δ), 0.2 mol/m3 (◊), 0.6 mol/m3 (Δ), 0.8 mol/m3 (○), 1.0 mol/m3 (□), and 2.92 mol/m3 (◊). (D) Final steady-state surface tension of aqueous 1-octanol as a function of environment solution concentration for Cdrop = 0.2 mol/m3 (◊), 0.4 mol/m3 (□), 0.6 mol/m3 (Δ), 0.8 mol/m3 (○), 1.0 mol/m3 (□), and 2.92 mol/m3 (◊). Solid lines represent theoretical predictions from Equation (1). (0.35 MB DOC) [file pone.0008281.s007.doc]

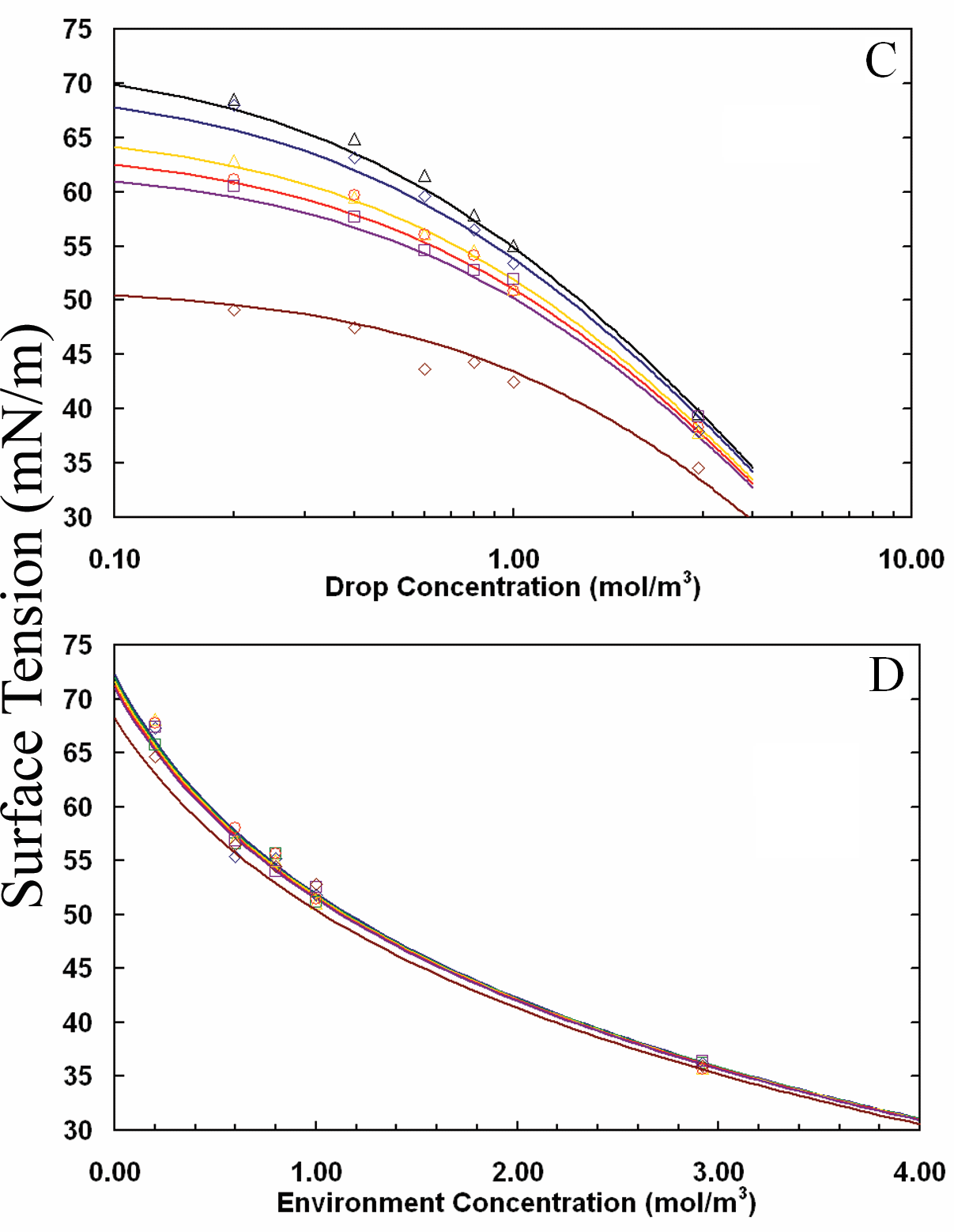


**Figure S5.** (A) Initial steady-state surface tension of aqueous 1-butanol as a function of drop solution concentration for C_env_ = 0 mol/m^3^ (**Δ**), 60 mol/m^3^ (□), 100 mol/m^3^ (Δ), and 400 mol/m^3^ (○). Note that (A) is a repeat plot of Figure 1 of Prpich AM, Biswas ME, Chen P [(2008) “Adsorption kinetics of aqueous n-alcohols: a new kinetic equation for surfactant transfer,” J. Phys. Chem. C 112: 2522-2528] and included here for comparison purposes. (B) Final steady-state surface tension of aqueous 1-butanol as a function of environment solution concentration for C_drop_ = 20 mol/m^3^ (**Δ**), 60 mol/m^3^ (□), 100 mol/m^3^ (Δ), and 400 mol/m^3^ (○). (C) Initial steady-state surface tension of aqueous 1-octanol as a function of drop solution concentration for C_env_ = 0 mol/m^3^ (**Δ**), 0.2 mol/m^3^ (◊), 0.6 mol/m^3^ (Δ), 0.8 mol/m^3^ (○), 1.0 mol/m^3^ (□), and 2.92 mol/m^3^ (◊). (D) Final steady-state surface tension of aqueous 1-octanol as a function of environment solution concentration for C_drop_ = 0.2 mol/m^3^ (◊), 0.4 mol/m^3^ (□), 0.6 mol/m^3^ (Δ), 0.8 mol/m^3^ (○), 1.0 mol/m^3^ (□), and 2.92 mol/m^3^ (◊). Solid lines represent theoretical predictions from Equation (1).
