# Supplementary material for: Tension at the Surface: Which Phase Is More Important, Liquid or Vapor?
Source: PLoS One. 2009 Dec 14;4(12):e8281. doi: 10.1371/journal.pone.0008281 (PMC2788621; doi:10.1371/journal.pone.0008281)
Supplement: Figure S6 — Aqueous 1-octanol dynamic surface tension profiles for consecutive drops from a continuous run using the same syringe and environment solution; Drop #1 (□), Drop #2 (◊). Drop solution concentration is 1.0 mol/m3 with pure water as the environment solution. (0.03 MB DOC) [file pone.0008281.s008.doc]

#

**Figure S6.** Aqueous 1-octanol dynamic surface tension profiles for consecutive drops from a continuous run using the same syringe and environment solution; Drop #1 (□), Drop #2 (◊). Drop solution concentration is 1.0 mol/m^3^ with pure water as the environment solution.
