# Supplementary material for: Tension at the Surface: Which Phase Is More Important, Liquid or Vapor?
Source: PLoS One. 2009 Dec 14;4(12):e8281. doi: 10.1371/journal.pone.0008281 (PMC2788621; doi:10.1371/journal.pone.0008281)
Supplement: Figure S7 — (A) Number density profiles of 1-butanol molecules. The values for hydroxyl hydrogen, hydroxyl oxygen and methyl carbon are depicted as black squares, red circles and green triangles, respectively. (B) Number density profiles of water molecules. The profiles connected using the black dotted line and the red solid line are for water hydrogen and oxygen, respectively. (0.60 MB DOC) [file pone.0008281.s009.doc]

#
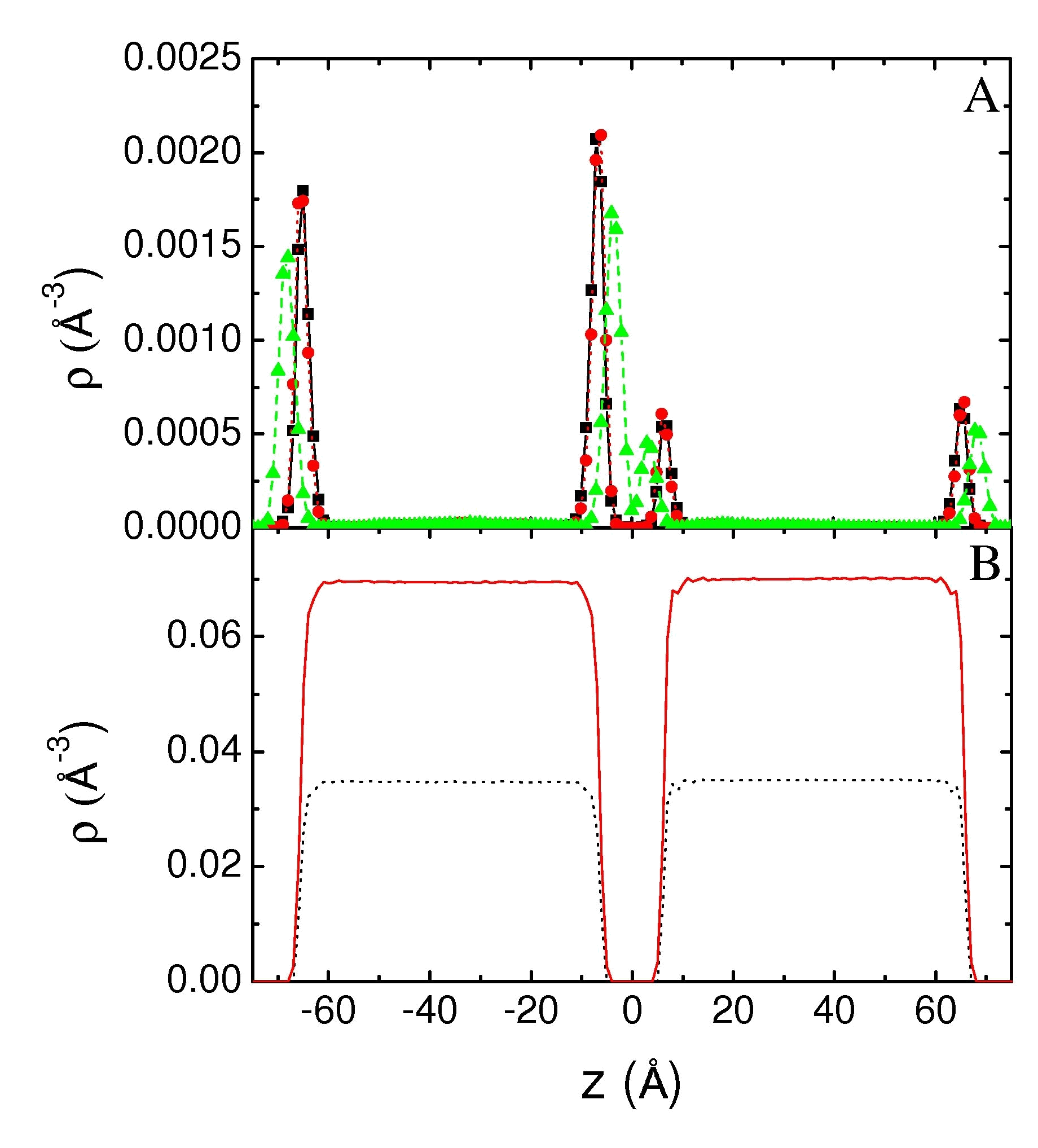


# Figure S7. (A) Number density profiles of 1-butanol molecules. The values for hydroxyl hydrogen, hydroxyl oxygen and methyl carbon are depicted as black squares, red circles and green triangles, respectively. (B) Number density profiles of water molecules. The profiles connected using the black dotted line and the red solid line are for water hydrogen and oxygen, respectively.
