# Supplementary material for: Tension at the Surface: Which Phase Is More Important, Liquid or Vapor?
Source: PLoS One. 2009 Dec 14;4(12):e8281. doi: 10.1371/journal.pone.0008281 (PMC2788621; doi:10.1371/journal.pone.0008281)
Supplement: Figure S8 — Hydroxyl oxygen - water hydrogen (a) and water oxygen - water hydrogen (b) radial distribution functions. The black, red and blue curves correspond to the molecules located at the Gibbs dividing surface (z = −6.27 Å), 4 Å (z = −10.27 Å) and 8 Å (z = −20.27 Å) away from the dividing surface into the liquid. Note that the hydrogen bonding H…O distance is typically 1.6∼2.0 Å, and the distance of the first peak in RDF is well within the hydrogen bonding distance. (0.33 MB DOC) [file pone.0008281.s010.doc]

#
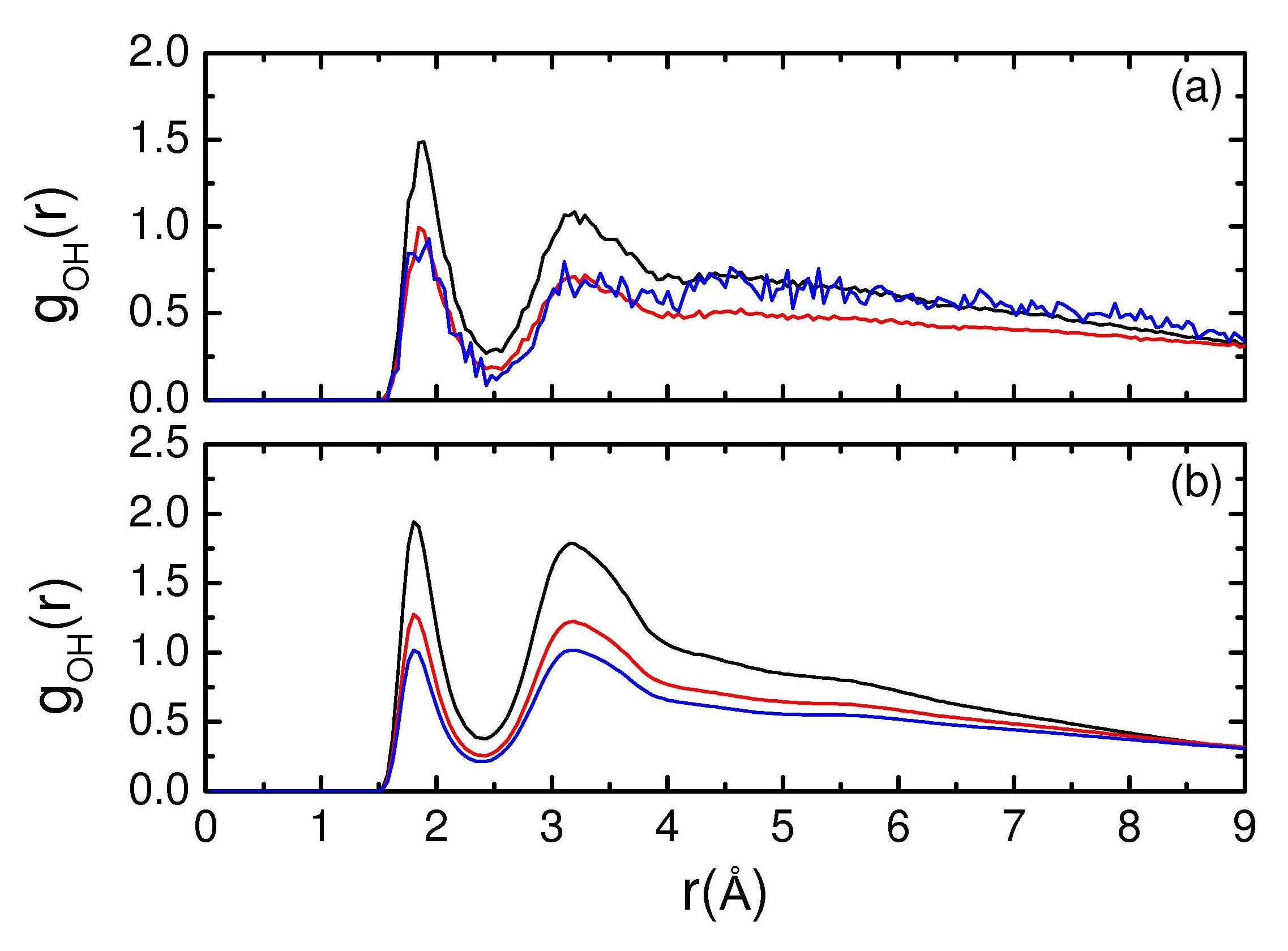


# Figure S8. Hydroxyl oxygen – water hydrogen (a) and water oxygen – water hydrogen (b) radial distribution functions. The black, red and blue curves correspond to the molecules located at the Gibbs dividing surface (*z =* -6.27 Å), 4 Å (*z =* -10.27 Å) and 8 Å (*z =* -20.27 Å) away from the dividing surface into the liquid. Note that the hydrogen bonding H...O distance is typically 1.6 ~ 2.0 Å, and the distance of the first peak in RDF is well within the hydrogen bonding distance.
